# Evaluation of the Analgesic Effect of High-Cannabidiol-Content Cannabis Extracts in Different Pain Models by Using Polymeric Micelles as Vehicles

**DOI:** 10.3390/molecules28114299

**Published:** 2023-05-24

**Authors:** Yoreny Román-Vargas, Julián David Porras-Arguello, Lucas Blandón-Naranjo, León Darío Pérez-Pérez, Dora María Benjumea

**Affiliations:** 1 Grupo de Toxinología y Alternativas Farmacéuticas y Alimentarias, Departamento de Farmacia, Facultad de Ciencias Farmacéuticas y Alimentarias, Universidad de Antioquia, Medellín 1226, Colombia; 2 Grupo de Investigación Macromoléculas, Departamento de Química, Facultad de Ciencias, Universidad Nacional de Colombia, Av. Carrera 30 # 45-03, Edif. 476, Bogotá 11001, Colombia; 3 Grupo Interdisciplinario de Estudios Moleculares-GIEM, Instituto de Química, Facultad de Ciencias Exactas y Naturales, Universidad de Antioquia, Medellín 1226, Colombia

**Keywords:** cannabis, cannabidiol, analgesic, polymer micelles, encapsulation

## Abstract

Currently, cannabis is considered an attractive option for the treatment of various diseases, including pain management. Thus, developing new analgesics is paramount for improving the health of people suffering from chronic pain. Safer natural derivatives such as cannabidiol (CBD) have shown excellent potential for the treatment of these diseases. This study aimed to evaluate the analgesic effect of a CBD-rich cannabis extract (CE) encapsulated in polymeric micelles (CBD/PMs) using different pain models. The PEG-PCL polymers were characterized by gel permeation chromatography and ^1^H-NMR spectroscopy. PMs were prepared by solvent evaporation and characterized by dynamic light scattering (DLS) and transmission electron microscopy. The analgesic activity of CBD/PMs and nonencapsulated CE rich in CBD (CE/CBD) was evaluated using mouse thermal, chemical, and mechanical pain models. The acute toxicity of the encapsulated CE was determined by oral administration in mice at a dose of 20 mg/kg for 14 days. The release of CBD from the nanoparticles was assessed in vitro using a dialysis experiment. CBD/PMs with an average hydrodynamic diameter of 63.8 nm obtained from a biocompatible polyethylene glycol-block-polycaprolactone copolymer were used as nanocarriers for the extract formulations with 9.2% CBD content, which corresponded with a high encapsulation efficiency of 99.9%. The results of the pharmacological assays indicated that orally administered CBD/PMs were safe and exerted a better analgesic effect than CE/CBD. The micelle formulation had a significant analgesic effect in a chemical pain model, reaching a percentage of analgesia of 42%. CE was successfully encapsulated in a nanocarrier, providing better stability. Moreover, it proved to be more efficient as a carrier for CBD release. The analgesic activity of CBD/PMs was higher than that of free CE, implying that encapsulation is an efficient strategy for improving stability and functionality. In conclusion, CBD/PMs could be promising therapeutics for pain management in the future.

## 1. Introduction

Chronic pain is an important health problem affecting approximately 8% of the world population [1]. This condition negatively impacts the physical and psychological health of sufferers, reducing their quality of life. Current pharmacological treatments do not effectively manage moderate and severe acute and chronic pain. In most cases, analgesics have insufficient activity, but the most effective analgesics produce serious adverse effects [2,3]. Some of the main medications prescribed for pain management are opioids, paracetamol, nonsteroidal anti-inflammatory drugs, muscle relaxants, anticonvulsants, antidepressants, and COX2 inhibitors [4]. The use of these drugs has undesirable side effects such as gastric or duodenal ulcers, renal toxicity, cardiovascular risk, constipation, sedation, somnolence, and fatigue. In the case of opioids, tolerance and dependence are common [5,6].

For centuries, cannabis has been used for medical purposes in different cultures. It is considered an attractive therapeutic option for the treatment of various diseases, including pain management [7,8,9]. More than 100 cannabinoid compounds have been identified. Among these, cannabidiol (CBD) and tetrahydrocannabinol (THC) are the major phytocannabinoids of pharmacological importance extracted from *Cannabis sativa* [10]. Current evidence on the analgesic effects of cannabis extracts and their compounds shows low efficacy, and not all studies have shown consistent results [11,12]. The analgesic efficacy of CBD and THC in the treatment of acute and chronic pain has been previously demonstrated in different animal models [7,13,14]. Numerous studies have evaluated the effect of isolated phytocannabinoids. However, some researchers have found that their activity is influenced by other compounds contained in the plant, promoting synergic interactions that enhance the pharmacological properties of cannabis [15,16]. For instance, CBD-rich cannabis extracts (CEs) produced a superior therapeutic profile for the treatment of refractory epilepsy compared to isolated CBD [17,18].

This enhanced performance is attributed to pharmacokinetic and pharmacodynamic interactions; thus, the therapeutic effects of whole-plant components exceed the sum of each individual part [19,20,21,22]. Despite the pharmacological potential of cannabis derivatives, their wide-ranging medical usage remains challenging. In particular, oral administration is problematic because of its unfavorable physicochemical characteristics. Phytocannabinoids are lipophilic molecules that are poorly soluble in aqueous media, susceptible to oxidation, and easily degraded by first-pass metabolism, thereby reducing their oral bioavailability [23,24].

The use of nanostructured vehicles has emerged as an option to overcome the above-mentioned limitations associated with cannabis derivatives. The encapsulation of essential oils extracted from hemp into alfalfa protein nanoparticles reportedly serves as an alternative to enhance their solubility in food applications [25]. Moreover, emulsion nanocarriers, hydrogels, emulgels, and lipid nanosystems have been used as CBD-release carriers [26,27]. Likewise, CBD could be microencapsulated with chitosan and cyclodextrins which can enable obtaining stable inclusion complexes [28,29,30].

When the main terpenes extracted from cannabis are encapsulated in PLGA and poly(ethylene glycol) (PEG)-poly (lactic-co-glycolic acid) (PLGA) nanoparticles, their in vitro analgesic activity increases [31,32]. Several nanostructured systems that provide some enhancement in bioavailability, particularly for nonpolar drugs, have been studied to obtain orally administered formulations. Polymeric micelles (PMs) are promising systems because they are highly stable colloidal dispersions that can comprise biodegradable and biocompatible amphiphilic copolymers. They are also capable of encapsulating lipophilic drugs and shielding them from degradation caused by substances found in physiological environments [24]. PMs are formed through the self-assembly of amphiphilic copolymers to obtain core–shell structures [33]. The hydrophobic core acts as a reservoir for hydrophobic drugs and modulates their release, increasing their circulation time and bioavailability [34]. The hydrophilic core provides water solubility and colloidal stability and regulates interactions with biological environments [35].

Among the polymeric materials investigated as nanocarriers for oral administration, poly(ε-caprolactone) (PCL) and PEG have attracted considerable interest [36,37]. PCL is a biocompatible, biodegradable, semicrystalline aliphatic polyester approved by the Food and Drug Administration. This polymer has been previously reported for the encapsulation and controlled release of multiple phytocannabinoids with extended therapeutic effects [38]. PCL degrades slowly with a minor alteration in pH, whereas other polymers, such as poly(lactic acid) (PLA), poly(glycolic acid) (PGA), and PLGA, induce acidification of the medium and could negatively affect the stability of cargo and tissues [33,34]. Meanwhile, a hydrophilic PEG coating endows nanoparticles with several advantages, such as stability in digestive fluids, poor adsorption, and interaction with plasma proteins and phagocytic cells, thereby avoiding premature elimination [39,40].

The present study aimed to investigate the in vivo analgesic activity of CBD/PMs using chemical, thermal, and mechanical pain models suitable for evaluating the efficacy of cannabis-derived formulations for the treatment of chronic pain. Pain is a complex sensory experience involving several signaling pathways that are activated and responsive to the noxious stimulus and the stimulus nature [41]. When intense heat, pressure, or chemical irritants are applied, primary sensory neurons are excited and transmit these stimuli as action potentials to the brain. In the brain, it is processed and translated into a sensation of pain. Several types of primary afferent fibers (nociceptors) in peripheral terminals respond to applied (chemical, thermal, or mechanical) stimuli [42]. To the best of our knowledge, this study is the first to characterize the in vivo analgesic effectiveness of a CBD-rich CE encapsulated in PMs and administrated orally.

## 2. Results

### 2.1. Preparation and Characterization of CBD/PMs

Amphiphilic block copolymers comprising biocompatible segments of mPEG and PCL [33,43] were used as precursors of micellar vehicles for a CBD-rich CE. The copolymers listed in Table 1 were synthesized by ROP starting from mPEG of 2 and 5 kDa as macroinitiators. 

In the first experiment, the effect of PCL and PEG length on the capacity of micellar vehicles to encapsulate CBD was studied using pure CBD, and the obtained values are listed in Table 1. The highest EE was achieved for the longest PEG and PCL segments. Studies have demonstrated that the length of PEG influences the interaction between the polymer nanoparticles and cells [44]. Short PEG chains allow coated nanoparticles to be up-taken by cells, whereas the interaction between long PEG segments and membrane proteins decreases. Therefore, the copolymer containing a PEG segment of 2.0 and 4.3 kDa of PCL was selected as the precursor for formulation development. The 1HRMN spectrum for PEG-b-PCL is presented in Figure S1 (Supplementary Materials), in which the characteristic signals for the monomer structures could be appreciated. Figure S2 (Supplementary Materials) also shows the chromatogram for hydrophilic PEG-b-PCL and its initiator m-PEG, in which the differences in retention time are clearly appreciated. This copolymer has a critical micelle concentration of approximately 5 mg/L.

The size of the polymer is not a significant factor because the vehicles are micelles, which are reversible aggregates of copolymer chains that form upon self-assembly in an aqueous medium, typically with sizes below 100 nm. The reported sizes correspond to equilibrium morphologies, and altering their sizes is challenging unless the composition of the copolymer or solvent is modified. According to the literature, particle size is critical for achieving high cellular internalization and enhanced diffusion through the gut mucous membrane [45].

Formulations from the CBD-rich extracts encapsulated in micelles obtained from the previously described copolymer were established using a modified nanoprecipitation methodology. The copolymer and the corresponding extract were dissolved in acetone and quickly mixed with distilled water. CBD/PMs were obtained after the evaporation of acetone at room temperature. The mass ratio of the organic phase to water was evaluated for a formulation with a fixed composition, and the data are provided in Table 2.

The results showed that when the volume of the organic phase equaled the amount of water, creaming of the oily compounds was observed, indicating poor encapsulation of the extract components. Meanwhile, a lower organic phase content produced more stable dispersions.

The hydrodynamic diameter of the particles, as measured by DLS, depended on the amount of water. When the water-to-organic phase ratio was as low as 10:1, the particles had the smallest diameter and narrow distribution. A decrease in the amount of water produced larger particles with minor variations in the PDIs. Using a large volume of water interfered with the recovery of extract-loaded nanoparticles and the adjustment of doses used in the toxic and analgesic tests. Accordingly, a 1:5 ratio of organic phase to water was selected to prepare the final formulation. In addition to obtaining a stable suspension, the particles were <100 nm in diameter and spherical, as shown in the TEM images showed in Figure S3 (Supplementary Materials). This formulation had a CBD content of 9.1 wt%, as determined by HPLC analysis.

**Figure 1:**
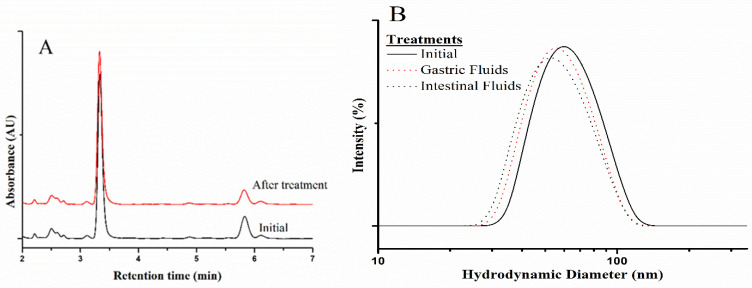
Evaluation of CBD/PMs in simulated gastrointestinal fluids. (**A**) Representative chromato-grams and (**B**) hydrodynamic-diameter distribution measured by DLS obtained before and after treating the formulation with gastric and intestinal fluids.

The stability of CBD/PMs against gastric fluids was analyzed by HPLC in comparison with the initial chromatographic profile obtained after immersing the formulation in simulated gastric and intestinal fluids at 37 °C. The results are shown in Figure 1. The peak associated with CBD did not change in intensity or retention time, indicating that this compound was stable in these media. The formulation exposed to the simulated fluids was further analyzed by DLS to determine any effect on the particle size, yielding the hydrodynamic diameter distributions compared in Figure 1. The given distributions indicated that the nanoparticle size was unaltered, corroborating that the micelles were stable in the evaluated media and thus exerted a shielding effect on the loaded extract.

**Figure 2:**
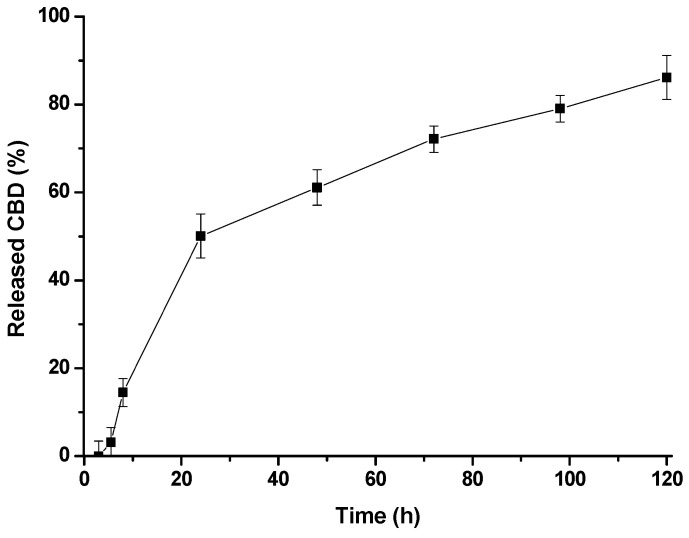
Release profile of CBD from CBD/PMs.

The ability of the micelle formulation to release the loaded extract was evaluated by measuring the amount of CBD that remained encapsulated in the micelles after dialyzing the formulations against medium under sink conditions (Figure 2). The release started after 3 h, indicating an induction period probably associated with the solvation of the micelle constituents, given their kinetically conditioned polymeric nature. According to the profile, the release rate depended on time and exhibited two regions. Initially, a high rate was observed; however, it decreased after 24 h, after which a constant slope was maintained for 120 h. Drug release from PMs has been postulated to be primarily governed by diffusion and micelle dissociation; however, given the polymeric nature of PMs, their dissociation is characterized by slow unimer exchange [46]. The initial stage, which showed faster CBD depletion, could be associated with a predominant diffusional mechanism. Conversely, the second mechanism depended on the micelle dynamics and probably involved molecules trapped in the polymer loops.

### 2.2. Acute Toxicity Test

The mice were orally administered CBD/PMs at a dose of 20 mg/kg and monitored for 14 days. During this period, no deaths or signs of toxicity were observed. Special attention was paid to signs such as animal behavior, sleepiness, respiration, and coloration of mucous membranes. Moreover, the administered dose did not cause weight loss, suggesting low toxicity. Body weight reduction is considered an indicator of deterioration in general mouse health status [47]. Likewise, mouse appetite was unaffected during the experiment. During the observation period, the animals had a constant consumption of water and food, causing a progressive increase in their weight. The values are presented in Table 3. The results showed that weight increased on days 1, 7, and 14. After sacrifice, no morphological changes were observed in the internal organs during the necropsy.

The weights of the liver, heart, kidneys, and spleen were comparable to the reference weights [48]. The values listed in Table 4 were compared using a two-tailed Student’s *t*-test for paired data, considering the reference values for each organ weight.

The dose of 20 mg/kg CBD/PM used in the toxicity test was selected based on a dose higher than that administered in all analgesia tests to ensure that the doses evaluated were not toxic. These findings are consistent with earlier reports on the safety of PEG-b-PCL in other systems [36,39]. Similarly, CBD has been presented as a safe substance in humans and animals, although further studies on these formulations are needed to corroborate their safety [25,40,41].

### 2.3. Evaluation of Analgesic Activity

#### 2.3.1. Tail-Flick Test

**Figure 3:**
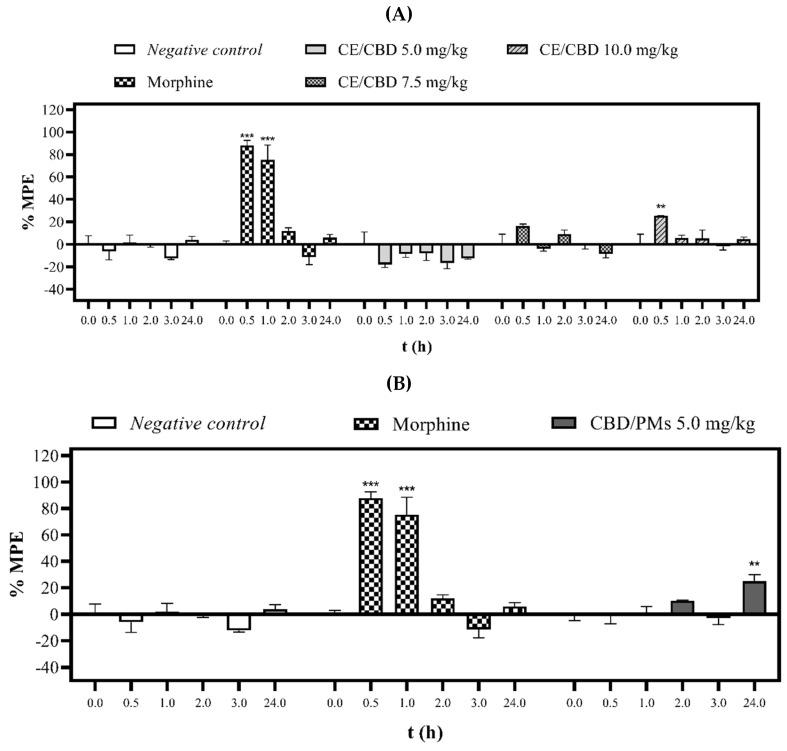
Antinociceptive effect of CE/CBD (**A**) and CBD/PMs (**B**) evaluated by tail-flick test. The values are expressed as the mean ± SEM for negative control, morphine (10.0 mg/kg), CE/CBD (5.0, 7.5, and 10.0 mg/kg, p.o.), and CBD/PMs (5.0 mg/kg, p.o.). For each experimental group, (*n* = 3). Significant difference in relation to the negative control group: (** *p* < 0.01, and *** *p* < 0.001) (two-way ANOVA followed by Tukey’s test).

Figure 3A,B show the analgesic effect of CE/CBD and CBD/PMs at different doses against thermal stimulation performed at 0.5, 1.0, 2.0, 3.0, and 24 h after oral administration of a single dose. The CE/CBD latency times were dose-dependent, showing a reduction in pain after oral administration (0.5 h) at doses of 7.5 and 10.0 mg/kg. The maximum possible analgesic effect percentages of 16% and 25% were statistically significant (*p* < 0.01) at the highest dose. However, the CE/CBD 5.0 mg/kg dose showed no effect during the 24 h of evaluation compared with the negative control. Conversely, CBD/PMs at a dose of 5.0 mg/kg (p.o.) maintained the analgesic effect at 24 h by 10%. The difference was significant (*p* < 0.001) compared with the negative control, which at the same time did not show an effect. Meanwhile, mice treated with negative control and morphine behaved as expected.

The results further indicated that CBD/PMs reduced the pain produced by the continuous application of noxious thermal stimuli to the mouse tail. Another significant finding was the better effect of CBD/PMS 24 h after oral administration than that obtained with morphine at the same time. This behavior confirmed that the CBD/PMs formulation released the active substances over 24 h, maintaining its analgesic effect. Conversely, morphine exerted an analgesic effect only 2.0 h after oral administration.

CE/CBD and CBD/PMs at doses of 10.0 and 5.0 mg/kg, respectively, presented similar analgesic potency but at different times. CE/CBD exhibited analgesic activity only at 0.5 h, whereas CBD/PMs showed a sustained analgesic effect at 24 h. Because CBD is a lipid-soluble substance [49], it is susceptible to first-pass metabolism through oxidation in phase I and glucuronidation in phase II [50]. This was consistent with our results, that is, the maximum plasmatic concentration of CBD was achieved 1 h after oral administration, explaining the result obtained for the raw extract [22,51]. In the case of CBD/PMs, the hydrophilic PEG shielded the CBD from the action of gastrointestinal fluids, and the small size of the particles increased the absorption of the extract constituents. Consequently, its blood circulation was favored, as indicated by its prolonged analgesic activity [40,52].

In our study, oral administration of CBD/PMs moderately changed the thermal pain threshold because of the type of behavioral response to pain. The tail-flick response, as a spinal reflex to thermal stimulation, measures the transient pattern of acute pain [53]. Recent studies have suggested the beneficial effects of CBD in the treatment of chronic pain [51]. An experiment found similar analgesia results with CBD oral solution at a dose of 5 mg/kg and cannabidiol-loaded mucoadhesive nanostructured lipid carriers for the treatment of neuropathic pain that produced a significant antinociceptive effect for more than 6 h [54]. This demonstrates that our cannabinoid delivery system was well designed to improve efficacy and prolong the duration of the analgesic effect. However, its analgesic efficacy remains debatable. Some reports have indicated that CBD by itself does not reduce pain and that it should be combined with THC. Conversely, other clinical studies have found that CBD alone has analgesic activity against neuropathic pain [55]. CBD can induce analgesic effects through different cellular pathways, including cannabinoid (CB1 and CB2) and TRPV1 receptors [56,57]. When thermal stimuli are applied, the TRPV1 receptor is activated. According to previous studies, CBD acts as an agonist of this receptor, causing the rapid desensitization of nociceptors. Receptor desensitization can render the TRPV1 channel nonfunctional to further painful stimulation [58]. CBD is a potent inhibitor of the hepatic metabolism of drugs through the inhibition of multiple isoenzymes of cytochrome P450 3A (CYP) [59]. It contributes to the analgesic effect of other cannabinoids in CE, such as THC [60,61].

#### 2.3.2. Electronic von Frey Test

**Figure 4:**
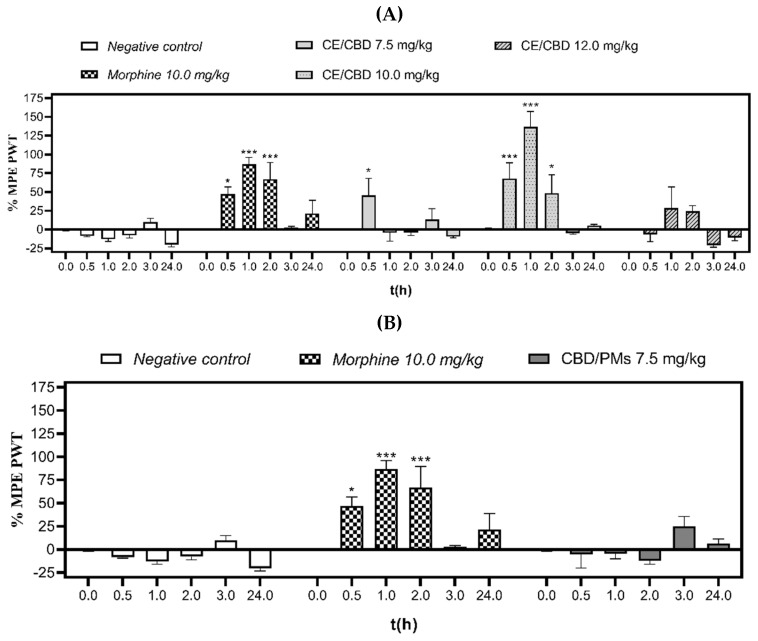
Antinociceptive effect of CE/CBD (**A**) and CBD/PMs (**B**) evaluated by electronic von Frey test. The values were expressed as the mean ± SEM. MPE%, percentage of maximum possible effect; PWT, paw-withdrawal threshold; time (h), application time of the mechanical stimulus (0.5, 1.0, 2.0, 3.0, and 24 h after p.o.) of all groups: negative control, morphine (10.0 mg/kg), CE/CBD (7.5, 10.0, and 12.0 mg/kg, p.o.), and CBD/PMs, (7.5 mg/kg, p.o.). For each experimental group, (*n* = 3). Significant difference in relation to the negative control group: (* *p* < 0.05 and *** *p* < 0.001) (two-way ANOVA followed by Tukey’s test).

After oral administration of three different doses of CE/CBD (7.5, 10.0, and 12.0 mg/kg) and a single dose of CBD/PM (7.5 mg/kg), the antinociceptive effect was evaluated by the electronic von Frey test at different times. According to the data provided in Figure 4A, at 0.5 h after oral administration of CE/CBD (7.5 and 10.0 mg/kg), we observed 45% and 68% reductions in PWT, respectively, and the differences were statistically significant (*p* < 0.05 and *p* < 0.001, respectively) compared with the negative control.

The dose of 10.0 mg/kg of CE/CBD significantly (*p* < 0.001) decreased allodynia at 1 h after oral administration with 137% protection. When the highest dose (12.0 mg/kg) was used, the lowest hindpaw PWTs were achieved. This trend indicated that the maximum analgesic effect was achieved with doses close to 10.0 mg/kg which weakened when the dose was decreased to 7.0 mg/kg and elevated to 12.0 mg/kg.

In the case of CBD/PMs Figure 4B, an induction time of 3 h was required to obtain an analgesic effect, which could be correlated with the in vitro release profile depicted in Figure 1. However, the in vivo interaction of polymer nanoparticles with biological molecules can alter their release rate. Figure 1 shows that at 24 h, the amount of CBD released was 50%, and sustained release occurred until 120 h. This trend, combined with the analgesic results, supports the need for tests with a higher CBD/PM dose and longer sampling periods [62]. Furthermore, cannabis extract with high levels of free cannabidiol exhibited a greater analgesic effect than CBD/PM because of the higher levels of THC and CBD in the free extract. Additionally, when encapsulating the extract, the loading capacity of CBD in the polymeric micelles was 9.2% (Figure S4a,b, Supplementary Materials).

However, orally administered CBD/PMs did not alter mechanical allodynia (*p* > 0.05) compared with CE/CBD. These findings were associated with reduced release of CBD in vivo, probably due to the hydrophobicity of the micelle core, the lipophilic properties of CBD limiting diffusion through the aqueous medium of the micelles, and additional resistance to CBD release. The PCL creates a hydrophobic barrier that limits access to water and the release of the compound [54].

The conduction of reflective responses to mechanical stimuli can be explained by the activation of polymodal C-nociceptors, which are complementary to Aδ fibers. Von Frey activates a different subset of sensory neurons (e.g., high-threshold and low-threshold mechanoreceptors) [63,64]. Research has shown that the analgesic effect occurs through TRPV1 receptors and that C-fibers are the primary mediators of reflective behaviors in tail-raise and paw-pinch tests [65]. The physicochemical characteristics of PMs favor absorption and the release of CBD from the polymeric matrix. As shown in the release profile of CBD at 24 h, it did not reach its maximum release; therefore, it is necessary to increase the dose of CBD in PMs and evaluate its analgesic effect at longer times [46].

#### 2.3.3. Phenylquinone-Induced Writhing Test

After 1 h of oral administration of CE/CBD (2.5, 5.0, and 10.0 mg/kg), results showed that pain was reduced by 37%, 43%, and 42%, respectively, compared with the negative control (*p* < 0.05). However, when the analgesic effect of CE/CBD (2.5 mg/kg) was evaluated 24 h after oral administration, no significant decrease in the number of abdominal contractions was found in mice compared with the negative control group, as shown in Table 5.

Notably, none of the studied doses exerted an analgesic effect similar to morphine. The maximum effect was presented with a dose of 5.0 mg/kg after 1 h of CE/CBD administration, reaching a significant percentage of pain protection of 43% (*p* < 0.001). Similar values were observed with the highest studied dose of CE/CBD, indicating that the maximum analgesic effect was reached at these doses, probably because of the total occupation of receptors.

CBD/PMs at a dose of 2.5 mg/kg showed a significant (*p* < 0.001) decrease in pain 24 h after oral administration, with 42% protection, indicating that the release of the active ingredient occurred in a controlled manner and was delayed compared with CE/CBD at the same measured time. CBD/PMs were evaluated 3 h after oral administration because, in the thermal pain model at that time, there were statistically significant differences (*p* < 0.001). Therefore, in the chemical pain model, the analgesic effect of CBD/PMs was evaluated 3 h after administration. This finding indicated that CBD/PMs had no analgesic effect because CBD was not released from the polymeric matrix at that time (Figure 2).

In contrast to mechanical stimulation, chemical contortion tests in mice evaluate responses to chemical irritation. They are the most extensively used standard tools for evaluating analgesics associated with partial or complete visceral distention [66]. Chemical stimulation activates various spinal signaling pathways, such as MAP kinases, PI(3)K, and spinal microglia. Therefore, the pain thresholds are different, and the doses for the types of pain also change [67]. The principal cannabinoid receptors (CB1 and CB2) can activate different signaling pathways through which cannabinoids can modulate the pain associated with chemical stimuli.

Several studies have found that abdominal contractions caused by the injection of chemical agents induce the release of kinins, cytokines, and prostaglandins (PGs). These phenomena are of particular importance, as PGE1, PGE2, and PGF2α have been shown to cause abdominal constriction in mice [68,69]. Finally, given that the encapsulated CE contained various cannabinoids, including THC, it is reportedly an effective antagonist of various analgesic agents, including phenylquinone. Thus, the low percentage of THC found in the extract may have contributed to the analgesic effect.

## 3. Materials and Methods

### 3.1. Drugs and Chemicals

A CE with a 1:29 ratio, that is, 31.65% CBD to 1.08% THC (CE/CBD), was supplied by an interdisciplinary group of molecular studies (GIEM; University of Antioquia, Colombia). ε-Caprolactone (CL; 98%), the HPLC chromatogram that can be seen in Figure S4a (Supplementary Materials) methoxy-PEG (mPEG; 2 kDa), pyrene (99%), tin (II) 2-ethyl hexanoate (Sn(Oct)_2_; 95%), a CBD standard solution (1.0 mg/mL in methanol), and other reagents and solvents used in the synthesis, purification, and characterization were purchased from Sigma–Aldrich. Before any synthetic procedure, toluene was dried by distillation over sodium and recollected in a balloon using molecular sieves and a nitrogen atmosphere. The CL and Sn(Oct)_2_ were dried using CaH_2_ and molecular sieves, respectively. PEG was dried by azeotropic distillation with toluene three times. CE/CBD was prepared in 2.0% (*w*/*v*) polysorbate 80 (Tween^®^ 80; Merck, Rahway, NJ, USA), 10% (*w*/*v*) propylene glycol (W. H. Curtin, USA), and 0.9% (*w*/*v*) saline (NaCl; Baxter, Colombia). The negative control (water) is a substance without pharmacological activity that accounts for the effect of the algesic substance that produces pain. Distilled water was used in this study. Morphine hydrochloride (morphine), used as a positive control, was obtained from the Special Administrative Unit of the National Narcotics Fund. Phenylquinone (Sigma–Aldrich) dissolved in saline and ethanol was used as the algesic agent. Acetonitrile, ethanol, and trifluoroacetic acid (>99.0%; Merck, USA) were used for the chromatographic analyses.

### 3.2. Synthesis and Characterization of PEG-Block-PCL Copolymer (PEG-b-PCL)

Block copolymers composed of PCL and PEG were synthesized. First, CL was polymerized by ring-opening polymerization (ROP) using mPEG as the initiator and Sn(Oct)_2_ as the catalyst. In a typical synthesis, mPEG (0.75 mmol) and Sn(Oct)_2_ (0.62 mmol) were dissolved in dried toluene under an argon atmosphere, followed by CL (32 mmol) addition. The reaction mixture was then stirred at 110 °C for 24 h. The reaction was stopped by the addition of cold diethyl ether to precipitate the polymer. The solid product was recovered via filtration and dried under reduced pressure at room temperature. In ^1^H-NMR (400 MHz, CDCl_3_, δ, ppm), the distinctive signals of PCL were as follows: 4.08 (t, *J* = 6.6), 2.33 (t, *J* = 6.6 Hz, 73H), 1.72–1.62 (m, 146H), 1.40 (m, 73H), and mPEG:3.66 (s, 494H).

The copolymers were characterized by ^1^H-NMR and GPC. The spectrum of copolymers was recorded on a 400 Ultrashield spectrometer operated at 400 MHz (Bruker, Mannheim, Germany) using CDCl3 as the solvent. The molecular weight distribution and polydispersity index (PDI) were determined by GPC using a high-performance liquid chromatography (HPLC) system (Thermoscitific, Ultimate 3000) equipped with a differential refractive index detector. Analyses were performed in THF at 0.8 mL/min flow rate in an HR 4E column. The calibration curve was constructed using polystyrene standards (2.5–50 kDa).

### 3.3. Preparation of CBD/PMs

The PMs were prepared by solvent evaporation. An organic phase comprising 320 mg of CE/CBD, 800 mg of PEG-b-PCL, and 32 g of acetone was thoroughly homogenized. Subsequently, the resulting solution was injected into 200 g of distilled water and stirred at room temperature until the acetone was completely evaporated.

### 3.4. Drug-Load Determination

The amount of CBD loaded into PMs was determined by an HPLC system (HPLC/UV LC-20 AT) using a chromatograph (Shimadzu^®^, Kyoto, Japan) equipped with a reverse-phase Infinity Lab Poroshell 120 EC-C18 column 4 µm (4.6 × 150 mm^2^) preceded by a security guard cartridge. UV absorption profiles were obtained using a diode array detector. The mobile phase consisted of water (eluent A) and acetonitrile containing 1 ppm TFA solution (eluent B). The flow rate and injection volumes were 1.2 mL/min and 20 μL, respectively. The elution gradient was set as follows: 0.0–5.0 min (0–70% B), 5.0–15.0 min (70–85% B), and 15.0–22.3 min (85% B). UV detection was performed at a wavelength of 215 nm (Figure S4b, Supplementary Materials).

In a typical sample preparation procedure, 5.0 mL of ethanol was added to 5.0 mg of the lyophilized particles. The mixture was sonicated for 10 min to disperse particles in ethanol. The mixture was centrifuged for 10 min at 9000 rpm to separate and remove the precipitated polymer. Finally, the drug solution was filtered (UltraCruz^®^ Syringe Filter, PVDF, 0.45 µm, 30 mm) and injected into the HPLC system for CBD detection. The amount of CBD loaded into the PMs (DL%) and entrapment efficiency (EE%) were calculated according to Equations (1) and (2).

(1) $$EE\;\left(\%\right)=\frac{CBD\;encapsulated}{Inicialamount\;of\;CBD}\times 100$$

(2) $$DL\;\left(\%\right)=\frac{CBD\;encapsulated}{Formulation\;mass}\times 100$$

### 3.5. CBD Release Assay

A 4 mL aliquot of encapsulated extract, corresponding to 1.6 mg of CBD, was added to a dialysis cassette (Thermo Fisher’s Slyde-A-Lizer [Waltham, MA, USA] equipped with a semipermeable membrane with a molecular weight cutoff of 7000 Da for proteins). The dispersion was dialyzed against 200 mL of PBS (pH 7.4) containing 1% Tween 80 at 37 °C. At regular intervals ranging from 1 to 120 h, 50 μL aliquots were taken from the cassette and diluted to 1 mL with methanol. The CBD content of the samples was quantified using HPLC. Changes in the corresponding peak integrals were monitored to assess drug release from the micelles.

### 3.6. Animals

Female Swiss albino mice (20–22 g) bred at the Bioterio of the University of Antioquia were used in all experiments. The reason for using only female mice was that a more significant perception of pain has been found. Therefore, sex differences influence measured analgesic effects [70,71]. All mice were maintained on a 12 h light/dark cycle (lights on from 06:00 to 18:00) in a temperature- and humidity-controlled facility with food and water available ad libitum, except during testing. For each pain model (chemical, thermal, and mechanical), three groups of mice were administered three different doses of CBD/PMs, a negative control group (water) of three mice was administered distilled water, and a positive control group of three mice was administered morphine. All animal procedures were reviewed and approved by the Ethics Committee for Animal Experimentation of the University of Antioquia (Protocol 117, dated April 25, 2019) and complied with the established guidelines of the Canadian Council on Animal Care and Public Health [72,73].

### 3.7. Acute Toxicity Test

In an acute toxicity study, female mice received a single oral dose of CBD/PMs (20 mg/kg). Guidelines suggest using a 2000 mg/kg dose in the absence of information about the substance tested, but research has been conducted on the individual safety of CBD [43,74] and PMs [33,75]. However, no studies have reported on the toxicity of CBD encapsulated in PEG-PCL PMs; therefore, a dose higher (20 mg/kg, p.o.) than that used in all analgesia tests (2.5, 5, 7.5, 10, and 12 mg/kg) was selected for acute toxicity assessment. The analgesic doses were selected based on those evaluated in studies on chronic pain [76,77]. The in vivo toxicological properties of CBD/PMs were examined according to the Organization for Economic Cooperation and Development (OECD-guideline) guideline 423 for acute toxicity tests [78]. A single 20 mg/kg dose of CBD/PM was administered orally on the first day. The animals were thoroughly observed during the initial 4h and monitored daily for 14 days. Each animal was examined and observed for changes in behavior and weight, food and water intake, signs of toxicity, and death. Toxicity signs, namely, alterations in the skin, fur, eyes, mucous membranes, and body weight and deaths, were recorded.

Immediately after sacrifice, the main internal organs, including the liver, kidneys, heart, and spleen, were evaluated macroscopically. Relative organ weights were expressed as percentages of body weight (BW%) (Equation (3)).

(3) $$BW\;\left(\%\right)=\left(\frac{eight\;of\;organ\;\left(g\right)}{body\;weight\;\left(g\right)}\right)\times 100$$

### 3.8. Tail-Flick Test

The thermal thresholds were measured using a tail-flick meter (PANLAB LE 7106; Spain). The tail-flick test evaluates nociceptive behavior [79] using a modified version of the method suggested by Meymandi [53]. This method was performed in four groups (*n* = 3): negative control, CE/CBD at three different doses (5.0, 7.5, and 10.0 mg/kg, p.o.), CBD/PMs (5.0 mg/kg, p.o.), and morphine (10.0 mg/kg, i.p.). Before performing the experiments, the thermal sensitivity of the animals to nociceptive stimuli was evaluated. The tails of each animal were placed in a slot to ensure precise positioning. Latency was recorded on a digital display as the time between holding the mouse’s tail in the groove and the heat-flux pain reaction, as indicated by the tail flick. Tail-flick meter latency was evaluated before and 30, 60, 120, 180 min, and 24 h after oral administration of CE/CBD, CBD/PMs, negative control, and morphine. The cut-off time was 12 s to prevent tissue damage. If the mice did not respond to the pain reaction within that time, it was recorded as 12 s. The results are expressed according to Equation (4) as the absolute time or percentage of maximum possible effect (MPE%).

(4) $$\%MPE\;\left(\%\right)=\left[\frac{\left(T_{1}-T_{0}\right)}{\left(T_{2}-T_{0}\right)}\right]\times 100$$

T_0_ was the latency time before different substances administration, and T_1_ was 30, 60, 120, or 180 min after different substances administration. Finally, T_2_ was the cut-off time.

### 3.9. Electronic von Frey Behavioral Test

Mechanical allodynia was assessed by measuring the paw-withdrawal threshold (PWT) using a von Frey automatic measuring electronic device (Cat. No. 38450, Ugo Basile^®^, Italy) with an adapted filament applied to the paw surface mice, which in turn measures the response threshold in grams [80,81].

Mice were individually placed in clear acrylic boxes (17 cm × 96 cm × 14 cm) divided into four spaces on a raised metal grid floor to facilitate paw access. Approximately 30 min before the test, all the animals were habituated to the equipment. Before oral administration of the substances, the filament was applied perpendicularly to the plantar surface of the hind paw. The force was gradually increased until the animal moved its paw away from the stimulus. Stimulation was performed in triplicates [82].

Animals were then divided into four treatment groups: CE/CBD (7.5, 10.0, and 12.0 mg/kg, p.o.), CBD/PMs (7.5 mg/kg, p.o.), negative control, and morphine (10 mg/kg, i.p.). PWTs were measured at 30, 60, 120, 180 min, and 24 h after the administration of the studied substances. The responses were obtained in grams, and the results are presented as MPE%.

### 3.10. Phenylquinone-Induced Writhing Test

The phenylquinone abdominal-writhing response in mice [83] was used to evaluate visceral nociception. Doses were prepared based on a concentration of CBD (CE/CBD) at 2.5, 5.0, and 10.0 mg/kg in a volume of 0.1 mL/10 g body weight and CBB/PMs at 2.5 mg/kg directly to the stomach by oral gavage. Mice were divided into eight groups (*n* = 3) including negative control (water) and morphine. The groups treated with CE/CBD (2.5, 5.0, and 10 mg/kg, p.o.) were injected 60 min after treatment with phenylquinone (1.6 mg/kg, i.p.). Animals treated with CBD/PMs (2.5 mg/kg, p.o.) and CE/CBD (2.5 mg/kg, p.o.) were injected with phenylquinone (1.6 mg/kg, i.p.) 24 h after treatment. Finally, the morphine group was injected with phenylquinone after 30 min of administrating 10.0 mg/kg, i.p. of this analgesic. Responses were measured based on writhing, characterized by contractions in the abdominal musculature followed by the extent of the hind limbs [84]. The analgesic effect is expressed as the pain-protection percentage (P%). P% was calculated as the average number of abdominal stretches in the control group minus the average number of abdominal stretches in the test groups and divided by the average number of abdominal contractions in the control group.

### 3.11. Statistical Analysis

The mean values and standard error of the mean (SEM) were calculated for all experimental results. For the phenylquinone and tail-flick tests, one-way and two-way ANOVA were used, respectively. For the electronic von Frey test, two-way analysis of variance (ANOVA) was used. Subsequent post hoc comparisons were performed using Tukey’s test. The accepted level of significance was set at *p* < 0.05. All data were analyzed using the GraphPad Prism 8 software.

## 4. Conclusions

Micelles obtained from PEG-b-PCL were used as vehicles for a CBD-rich extract, resulting in the generation of nanometric particles with a CBD loading of 9.1%. The loaded micelles exhibited controlled release of CBD for 24 h and provided stability against simulated gastric fluids. The developed CBD/PM formulation was safely administered orally to experimental animals. Furthermore, CBD/PMs maintained the analgesic effect for a longer period than CE/CBD in the three pain models studied, namely, chemical, thermal, and mechanical. Both CE/CBD and CBD/PMs showed better analgesic effects in the application of a chemical stimulus than in the application of thermal and mechanical stimuli. In the thermal pain model, CBD/PMs showed a stronger analgesic effect than CE/CBD. CBD/PMs also reduced thermal hyperalgesia and mechanical allodynia in these acute pain models. These results demonstrated that encapsulation of cannabinoids in micelles protected them from the physiological environment. Maintaining analgesic effects. Owing to its increased effectiveness, this formulation could be used for the treatment of chronic pain, although long-term safety studies are needed to evaluate the effects of chronic administration of CBD/PMs. Additionally, micelles could serve as a basis for future research on the less studied phytocannabinoids regarding their applications in pain management and other therapeutic uses.

## Figures and Tables

**Table 1:** Relationship between molecular weight and encapsulation efficiency of PEG-PCL with CBD. PEG, polyethylene glycol; PCL Mn NMR-H, number-average molecular weight Mn of poly ε-caprolactone (PCL) measured by proton nuclear magnetic resonance; GPC, gel-permeation chromatography; EE%, encapsulation efficiency; D, dispersity; D_h_ (nm), particle diameter; PDI, polydispersity index.

PEG (Mn) kDa	PCL Mn (NMR-H) kDa	GPC (Mn) kDa	D (GPC)	EE% (CBD)	D_h_ (nm)	PDI
2.0	1.87	4.34	1.20	91.92	83.00	0.27
2.0	4.31	5.50	1.17	97.66	66.50	0.21
5.0	4.00	11.8	1.21	98.31	52.60	0.23

**Table 2:** Characterization of CBD/PEG-b-PCL obtained under different mass ratios of organic phase to water. D_h_ (nm), particle diameter; PDI, polydispersity index.

Mass Ratio of Organic Phase to Water	D_h_ (nm)	PDI	Physical Appearance
1:10	44.66 ± 0.97	0.21 ± 0.01	Homogenous suspension
1:5	86.74 ± 2.41	0.22 ± 0.01	Homogenous suspension
1:3	118.85 ± 3.50	0.25 ± 0.01	Homogenous suspension
1:1	94.04 ± 1.65	0.19 ± 0.02	Phase separation

**Table 3:** Physiological parameters: increase in body weight and consumption of mice treated orally with a CBD/PMs. Data are expressed as the mean ± standard error of the mean; *n* = 3.

Sex	CBD/PMs Dose (mg/kg)	Body Weight (g)			Dead/Total (Number)	Symptoms
		1st Day	7th Day	14th Day		
Female	20.0	19.6 ± 0.3	21.0 ± 0.5	21.5 ± 0.6	0/3	None

**Table 4:** Effect of a single oral administration of CBD/PMs on the relative organ weights of females (g/100 g of body weight).

Organs	BW% (Mean ± SEM)	*p*-Value ^1^
Liver	4.98 ± 0.10	0.09
Kidneys	1.65 ± 0.01	0.11
Heart	0.70 ± 0.09	0.84
Spleen	0.48 ± 0.02	0.17

^1^ Student’s *t*-test, α = 0.05; significant differences are indicated for *p* < 0.05 compared with the reference data [48].

**Table 5:** Antinociceptive effect of CE/CBD and CBD/PMs. N, number of abdominal writhes induced by phenylquinone; P%, pain protection percentage; negative control; morphine 10.0 mg/kg; CE/CBD (2.5, 5.0, and 10.0 mg/kg, p.o.); and CBD/PMs (5.0 mg/kg).

			CE/CBD				CBD/PMs	
		Morphine	1 (h)			24 (h)	3 (h)	24 (h)
Measures	Negative Control	10.0 mg/kg	2.5 mg/kg	5.0 mg/kg	10.0 mg/kg	2.5 mg/kg	2.5 mg/kg	2.5 mg/kg
N	45 ± 2	0 ± 0 ***	29 ± 1 **	26 ± 3 ***	26 ± 1 ***	36 ± 2	31 ± 4	24 ± 5 ***
*p*%	0 ± 4	100 ± 0 ***	37 ± 2 **	43 ± 6 ***	42 ± 3 ***	8 ± 5	8 ± 12	42 ± 13 ***

For each experimental group, (*n* = 3). Significant difference in relation to the negative control group: (** *p* < 0.01 and *** *p* < 0.001) (one-way ANOVA followed by Tukey’s test). Values are expressed as the mean ± SEM.

## Data Availability

Not applicable.

## Supplementary Materials

The following supporting information can be downloaded at: https://www.mdpi.com/article/10.3390/molecules28114299/s1, Figure S1: 1HNMR spectrum of PEG-b-PCL used as precursor for the micellar vehicles; Figure S2: GPC Chromatogram of amphiphilic PEG-b-PCL and its initiator m-PEG; Figure S3: Characterization of formulation CBD/PMs by TEM. The images show two different magnifications; Figure S4 (a). HPLC chromatogram for the cannabis extract before encapsulation. Quantitative amounts of THC: 1.08%, and CBD: 31.65%; Figure S4 (b). HPLC chromatogram for the encapsulates cannabis extract. Quantitative amounts of: CBD: 9.2%.
