# Supplementary material for: Evaluation of the Analgesic Effect of High-Cannabidiol-Content Cannabis Extracts in Different Pain Models by Using Polymeric Micelles as Vehicles
Source: Molecules. 2023 May 24;28(11):4299. doi: 10.3390/molecules28114299 (PMC10254120; doi:10.3390/molecules28114299)
Supplement: Supplementary file 1 [file molecules-28-04299-s001.zip › molecules-2272359-supplementary.pdf]

Supplementary Materials

Evaluation of the Analgesic Effect of High-cannabidiol-content Cannabis Extracts in Different Pain Models by Using Polymeric Micelles as Vehicles

Yoreny Román-Vargas [1,*], Julián David Porras-Arguello [2], Lucas Blandón-Naranjo [3], León Darío Pérez-Pérez [2] and Dora María Benjumea [1,*]

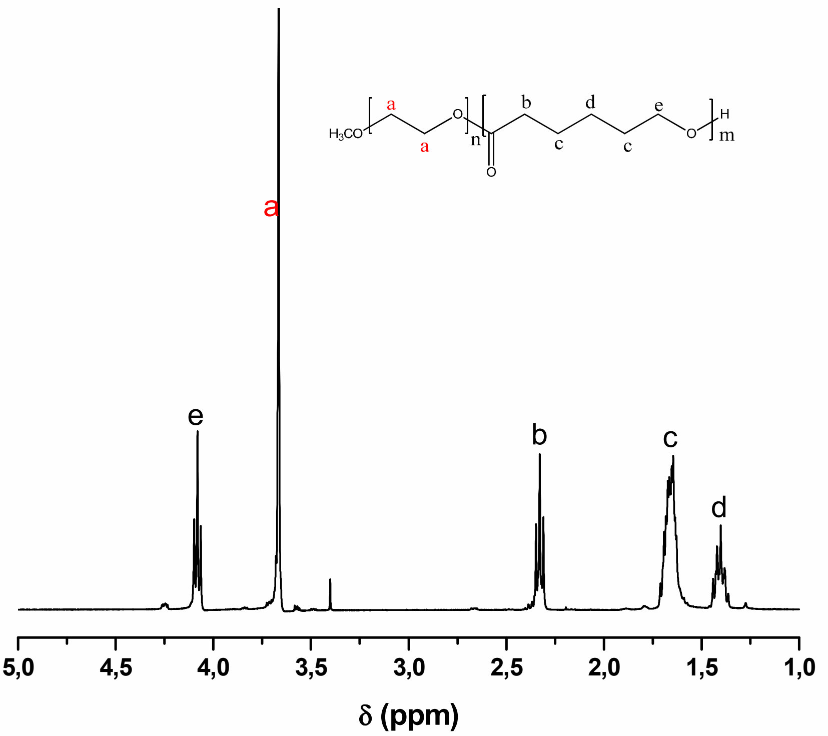

Figure S1. ^{1}HNMR spectrum of PEG-b-PCL used as precursor for the micellar vehicles.

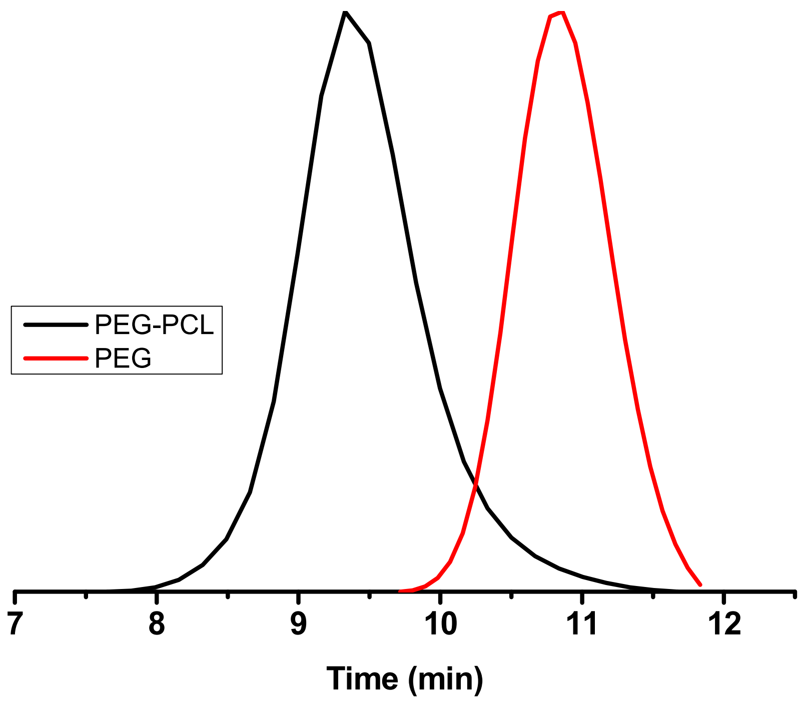

Figure S2. GPC Chromatogram of amphiphilic PEG-b-PCL and its initiator m-PEG.

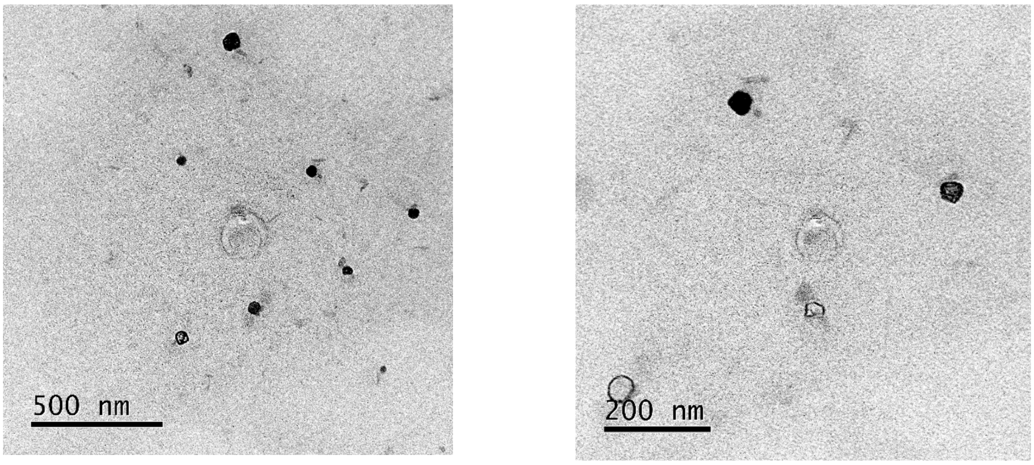

Figure S3. Characterization of formulation CBD/PMs by TEM. The images show two different magnifications.

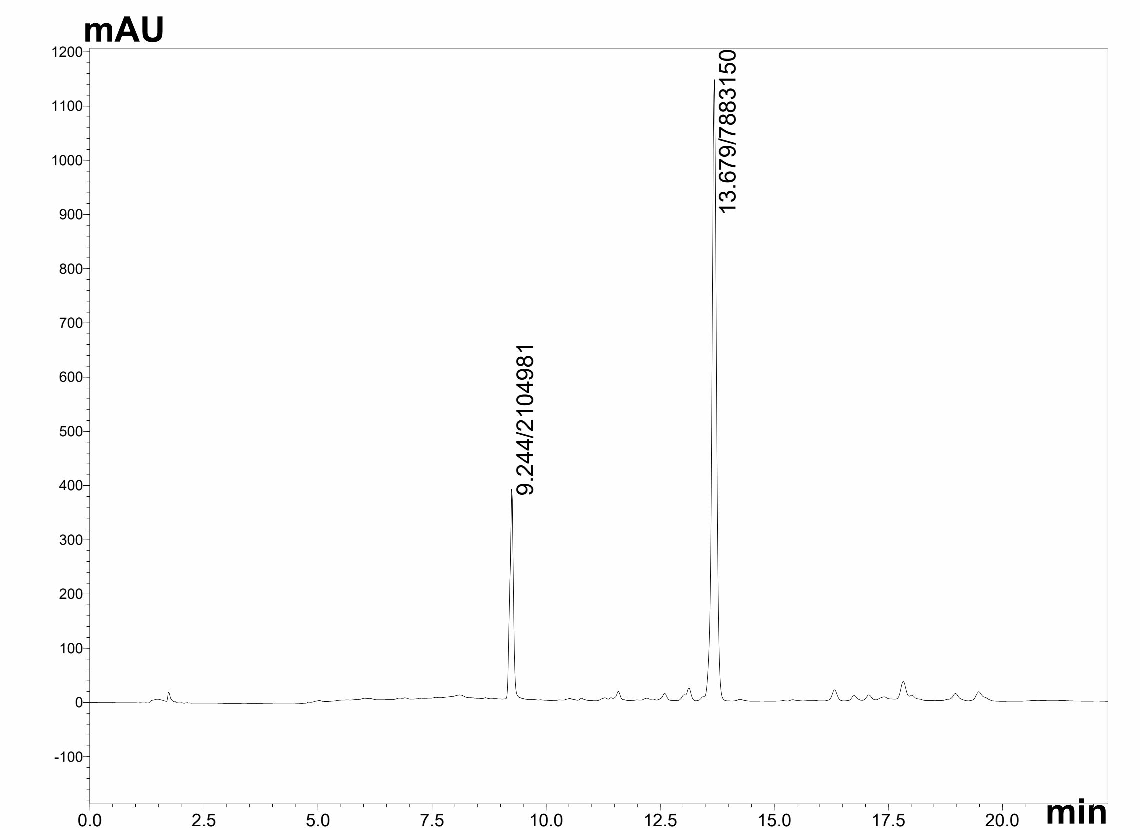

Figure S4 (a). HPLC chromatogram for the cannabis extract before encapsulation. Quantitative amounts of THC: 1.08%, and CBD: 31.65%.

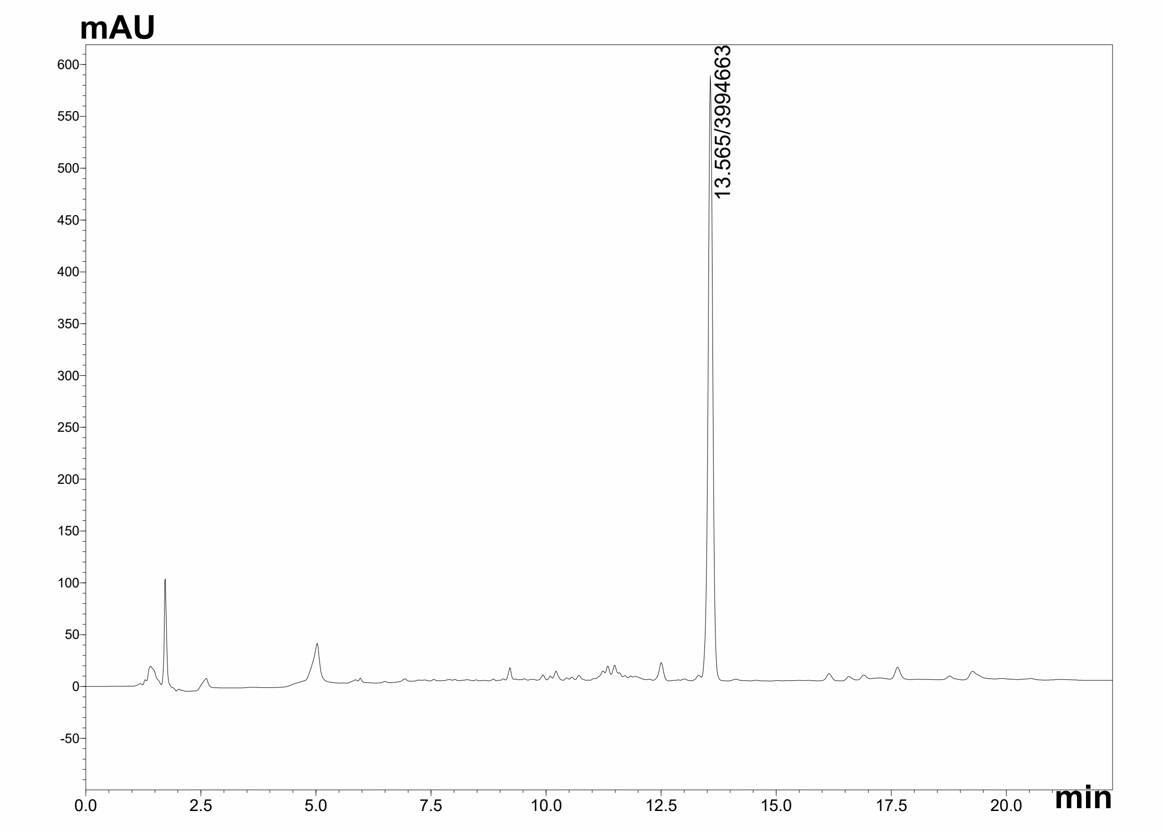

Figure S4 (b). HPLC chromatogram for the encapsulates cannabis extract. Quantitative amounts of: CBD: 9.2%
